# MRI changes of adjacent segments after transforaminal lumbar interbody fusion (TLIF) and foraminal endoscopy: A case–control study

**DOI:** 10.1097/MD.0000000000031093

**Published:** 2022-10-14

**Authors:** Shunmin Wang, Deyu Yang, Gengyang Zheng, Jie Cao, Feng Zhao, Jiangang Shi, Ruijin You

**Affiliations:** a 910 Hospital of China Joint Logistics Support Force, Fujian, PR China; b Department of Orthopedic Surgery, Spine Center, Changzheng Hospital, Second Military Medical University, Shanghai, PR China.

**Keywords:** adjacent segment degeneration, endoscopic surgery, LDH, TLIF

## Abstract

Intervertebral foramen endoscopy has developed rapidly, but compared with transforaminal lumbar interbody fusion (TLIF), the progress of degeneration is unknown. We aim to compare the changes of intervertebral disc and intervertebral foramen in adjacent segments after TLIF and endoscopic discectomy for patients with lumbar disc herniation (LDH).

**Methods:**

From 2014 to 2017, 87 patients who were diagnosed with single-level LDH and received surgery of TLIF (group T, n = 43) or endoscopic discectomy (group F, n = 44) were retrospectively analyzed. X-ray, MRI, CT and clinical symptoms were recorded before operation and at the last follow-up (FU). The neurological function was originally evaluated by the Japanese Orthopaedic Association (JOA) scores. Radiological evaluation included the height of intervertebral space (HIS), intervertebral foramen height (FH), intervertebral foramen area (FA), lumbar lordosis (CA) and intervertebral disc degeneration Pfirrmann scores.

**Results:**

There was no significant difference in baseline characteristics, JOA improvement rate, reoperation rate and complications between the two groups. The age, average blood loss, average hospital stays and average operation time in group F were lower than those in group T. During the last FU, HIS, CA and FA decreased in both groups, and the changes in group T were more significant than those in group F (*P* < .05). There was no significant difference in FH changes between the two groups (*P* > .05).

**Conclusion:**

Both TLIF and endoscopic surgery can achieve good results in the treatment of LDH, but the risk of lumbar disc height loss and intervertebral foramina reduction in the adjacent segment after endoscopic surgery is lower.

## 1. Introduction

Disc changes in the adjacent segment after lumbar fusion has aroused full attention, and the relationship between the two has been reported in many literatures.^[1]^ adjacent segmental degeneration (ASD) is defined as degeneration of adjacent intervertebral disc after surgery by observation on imaging, regardless of symptoms.^[2]^ In recent years, endoscopic technology has been widely used in clinic. Compared with open surgery, for example transforaminal lumbar interbody fusion (TLIF), endoscopic surgery has advantages of less trauma, less postoperative pain and a more rapid recovery, and the curative effect is equivalent to that of open surgery.^[3,4]^ However, there is a lack of systematic comparative study focusing on ASD after endoscopic surgery and TLIF. Although various novel operations may help to attenuate ASD (especially for motion-preserving surgery),^[1,5]^ their efficacies are still controversial. The purpose of this study was to compare the effects of endoscopic discectomy and TLIF on the changes of adjacent segmental disc height and foraminal changes, to review the literature to analyze its risk factors, and to raise awareness of ASD.

## 2. Methods

The study has been approved by the hospital’s board of directors and informed consent through institutional review, including details of the operation, including treatment mechanisms, predicted outcomes, and potential risks and adverse effects.

### 2.1. Patients’ population

Patients diagnosed with single-segment LDH and undergoing TLIF or endoscopic discectomy at our institution from June 2014 to June 2017 were included in this study. Inclusion: clinical diagnosis of L5/S1 LDH and complete imaging data. Exclusion: multi-segmental LDH, scoliosis, fracture, slippage and other lumbar spine diseases. (2) Unable to undergo surgery. (3) History of previous lumbar spine surgery. Group F: nerve compression leading to symptoms such as low back pain and intermittent claudication; ineffective conservative treatment: lumbar disc herniation (LDH), prolapse, free: symptoms cannot be relieved and continue to worsen: LDH with lateral saphenous fossa or local spinal stenosis, etc. Group T: the above criteria were accompanied by severe muscle weakness, foot drop, cauda equina syndrome, etc. endoscopic discectomy was difficult. Patients with osteoporosis in both groups continued pharmacological treatment.

### 2.2. Surgical procedure for LDH

TLIF (T group): endotracheal intubation under general anesthesia, the patient took a prone position and raised the waist bridge. A 10‐12 cm median incision was performed and peeled off layer by layer along the bilateral sub-periosteum of the spinous process.

Fully expose the lamina and articular process. The inferior articular process and part of the upper joint. Chisel off the process and hyperplastic osteophyte and bite off the ligamentum flavum, such as bilateral symptoms. If it is heavy, the contralateral decompression is carried out in the same way. Cage rack placement and connection of titanium. Place cage rack and connect titanium. Indwelling 1 negative pressure drainage tube. Suture incision layer by layer.

Foraminal endoscopic discectomy (F group): prone position, chest and ilium cushion soft pillow raised to make the abdomen empty, fully expand the intervertebral foramen and reduce the intervertebral foramen plastic operation. Determine the puncture path: Mark the outline of the ilium and determine the hand under fluoroscopy. The operative segment, and then determine the puncture distance according to the patient’s body size, in order to match the vertebrae. The horizontal gap is marked by a diagonal line with an angle of about 30°, and the puncture point is the line and the distance. The point of intersection of parallel lines at a predetermined distance from the rear median line. Disinfect and spread towels. After that, the local anesthetic diluted to 1% was applied to the skin and subcutaneous of the puncture point. Fascia infiltration anesthesia, and then the 18G puncture needle was punctured slowly until there was obvious obstruction force, that is, at the fascia of the lumbar dorsal muscle, the puncture needle is slightly retracted and blocked by local anesthesia. Continue to deepen the puncture needle to the tip of the superior articular process and replace 0.5% lidocaine. Due to the anesthesia of the facet joint, the puncture needle was withdrawn slightly to increase and deepen the tilt of the head. Through the safety triangle puncture along the direction of the spinal canal, it is confirmed that the needle tip is located in the right position. The midline of the spinous process is connected with the posterior edge of the vertebral body laterally. After cutting the skin with a sharp knife, insert it. Enter the guide wire, then use the step-by-step sleeve to expand the soft tissue, and then the fourth-stage ring. Saw to enlarge the intervertebral foramen step by step (each step is done under fluoroscopy, ring saw. Do not exceed the inner edge of the pedicle), and finally the working sleeve is placed smoothly and the fluoroscopy is accurate. It is recognized that it is located at the predetermined target position. Turn on the imaging system and carefully identify. Microscopic structure, separation and adhesion, removal of protruding nucleus pulposus tissue, surrounding. Decompression of the walking nerve root and detection of the pressure of the superior exit nerve root. Until the nerve root pulses with the pulse can be seen under the microscope, and the fibers are treated by radiofrequency thermocoagulation. The ring is formed, and the skin is sutured after careful hemostasis.

### 2.3. Clinical and radiological assessment

Demographics information including age, gender, duration of symptoms, body mass index, osteoporosis, blood loss, operation time and length of stay were evaluated to between groups. Patients were followed up for at least 36 months after surgery.

Radiologic data include the following parameters:

1. The Cobb angle (CA) of the whole lumbar lordosis: the angle between the line at the upper endplate of L1 and the upper endplate of S1.
2. Cross-sectional area (FA) and height (FH) of intervertebral foramen: on the sagittal section of the intervertebral foramen, the line around the corresponding intervertebral foramen on the sagittal section forms an area and the height of the upper and lower edges.^[6]^
3. The height of the anterior intervertebral space (AH).
4. The height of the middle intervertebral space (MH).
5. The height of the posterior intervertebral space (PH).
6. Pfirrmann grade: intervertebral disc degeneration was evaluated by Pfirrmann grade.
7. Nervous system function was obtained using Japanese Orthopedic Association Scores System (JOA Scores).
8. Alleviation of original symptoms, re-operation rates, complications were counted.

The data of preoperative and last follow-up (FU) were measured in all patients.

### 2.4. Statistical analysis

Statistical analysis was performed using the Statistical Package for the Social Sciences version 23.0 (IBM Armonls, NY, USA). Continuous variables were recorded as mean values ± standard deviation (SD), and categorical variables were expressed by proportions (%). The unpaired 2-tailed Student *t* test or Mann–Whitney *U* test were performed to compare the mean values or data distribution of continuous variables. And categorical variables were compared with the *χ*2 (Chi-square) test or Fisher exact test, as appropriate. The data measured before and after the last FU were statistically analyzed. Paired *t*-test was used for the last comparison before operation, and independent sample *t*-test was used for the comparison between groups. A *P* value of <.05 was considered statistically significant.

## 3. Results

A total of 87 patients were enrolled in our study the patients were divided into F group (n = 44) and T group(n = 43), including 24 males and 20 females in Foraminal group with an average age of 51.75 ± 3.65 years and 25 males and 18 females in TLIF group with an average age of 53.89 ± 5.21 years. The demographic characteristics of patients were summarized in Table 1 and baseline characteristics were well balanced between the two groups, including gender, and basic physical condition. But the age, blood loss, operation time, length of stays in group F were lower than those in group T(*P* < .05).

**Table 1:** Baseline demographic information of patients with LDH. LDH = lumbar disc herniation.

Variable	F group (n = 44)	T group(n = 43)	*P* value
Age	51.75 ± 3.65	53.89 ± 5.21	.029
Female	20 (45.45%)	18 (41.86%)	.735
Duration of symptoms	22.57 ± 5.36	21.98 ± 4.47	.579
BMI	20.48 ± 2.85	21.05 ± 2.77	.347
Osteoporosis	15(34.09%)	13(30.23%)	.700
Blood loss (mL)	145.30 ± 10.80	20.35 ± 9.87	<.001
Operation time (min)	64.65 ± 11.03	77.65 ± 7.80	<.001
Length of stay (days)	1.58 ± 0.22	4.18 ± 0.73	<.001

**Figure 1.**
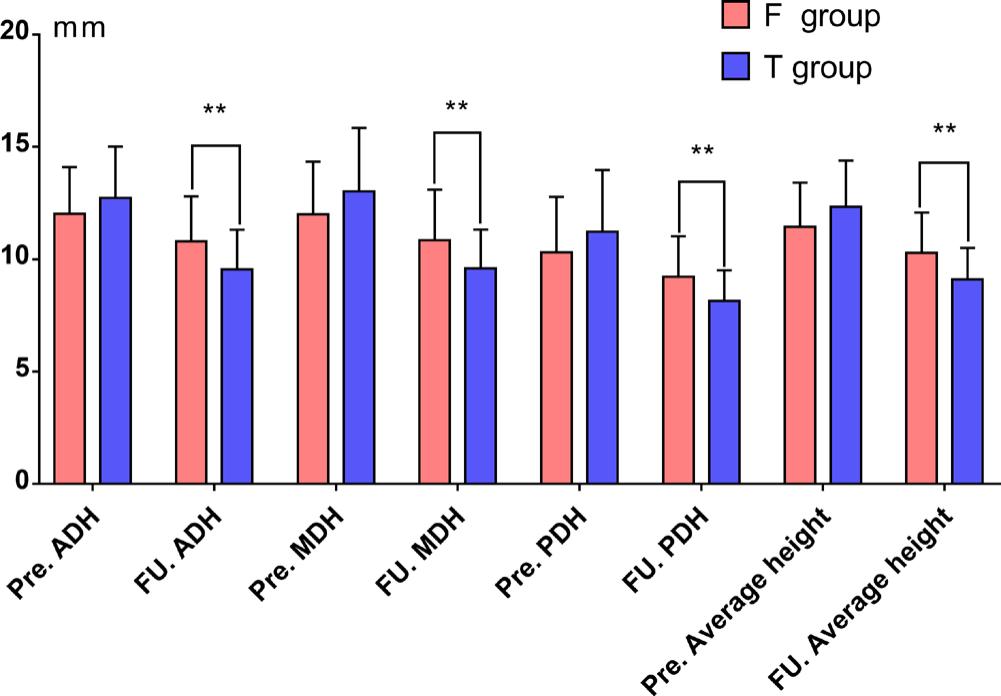
Change of the HIS of patients in the two groups HIS = height of intervertebral space.

The date of the height of intervertebral space (HIS) is summarized in Table 2. Before surgery, there was no statistical significance between group F and group T regarding AH, MH, and PH. but the mean HIS was significantly higher in group T. At the final FU the mean HIS decreased in two group (*P* < .05). Noticeably, the change of AH, MH, PH and average height in group F was all lower than that in group T (all *P* < .05) (Fig. 1).

**Table 2:** Change of the HIS of patients in the two groups.

Variable	F group (n = 44)	T group (n = 43)	*P* value
mean ± SD, mm			
Pre. ADH	12.02 ± 2.09	12.74 ± 2.28	.128
FU. ADH	10.81 ± 1.99*	9.55 ± 1.77*	.003^#^
Change. ADH	1.21 ± 0.73	3.19 ± 2.13	<.001^#^
Pre. MDH	12.01 ± 2.33	13.02 ± 2.83	.072
FU. MDH	10.86 ± 2.24*	9.60 ± 1.73*	.004^#^
Change. MDH	1.15 ± 1.14	3.43 ± 2.35	<.001^#^
Pre. PDH	10.31 ± 2.47	11.24 ± 2.73	.099
FU. PDH	9.24 ± 1.79*	8.16 ± 1.36*	.002^#^
Change. PDH	1.07 ± 1.45	3.07 ± 1.97	<.001^#^
Pre. average height	11.45 ± 1.96	12.33 ± 2.07	.045^#^
FU. average height	10.30 ± 1.79*	9.10 ± 1.41*	<.001^#^
Change. average height	1.14 ± 0.70	3.23 ± 1.37	<.001^#^

ADH = anterior disc height, MDH = middle disc height, PDH = posterior disc height; the average value of the three heights.

#Comparison of parameters between the two groups.

* *P* < .05, comparison of parameters within the same groups before surgery and final FU.

**Figure 2.**
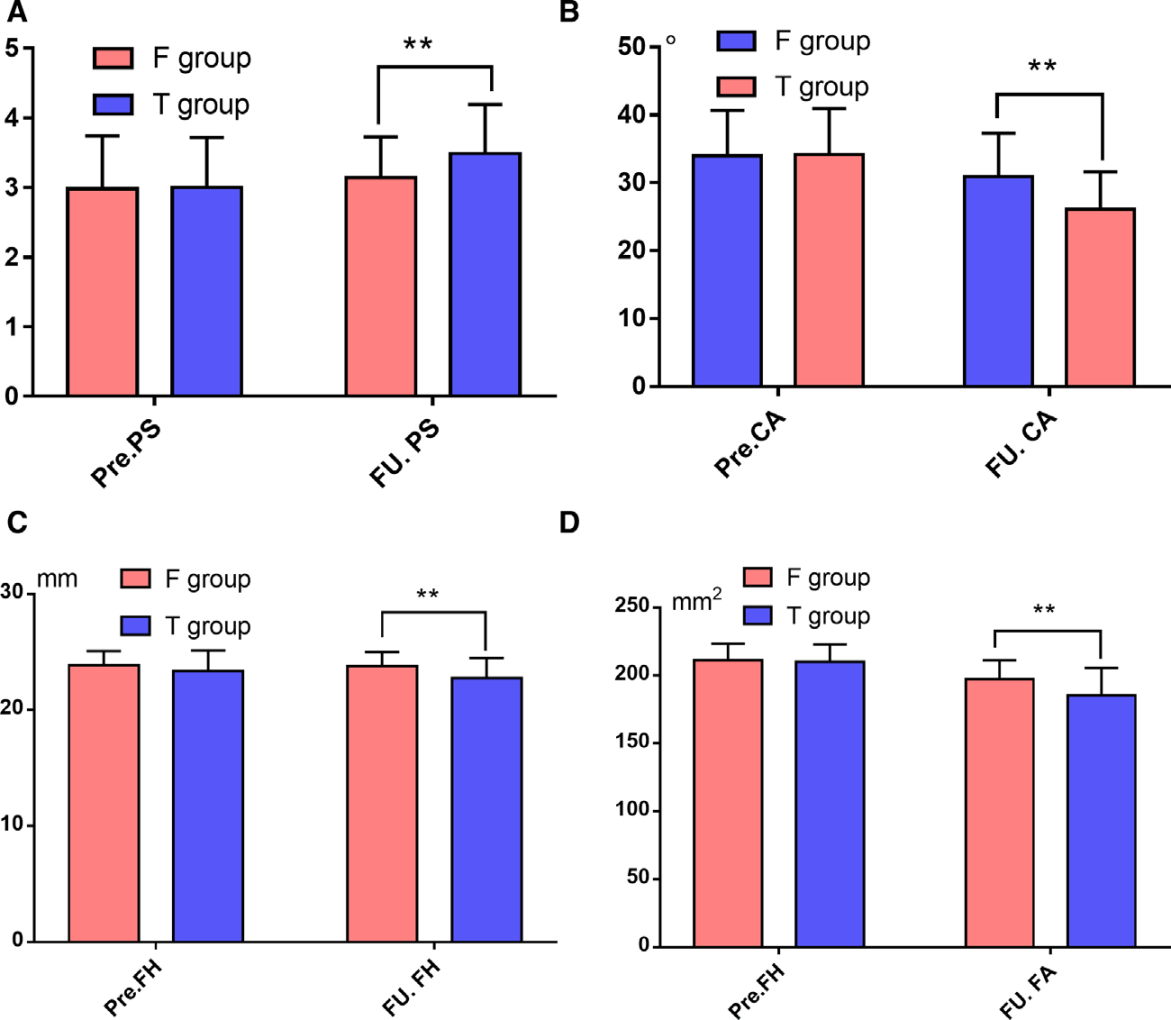
Imaging outcomes of patients in the two groups.

Table 3 demonstrates the changes of imaging outcomes between the two groups. We did not find any statistical difference in the comparison of FH, Pfirrmann scores, CA and FA within the two groups before surgery (all *P* > .05). However, the change of Pfirrmann scores, CA, and FA was significantly larger in group T at the last FU (*P* < .05). Although there is a difference of the reduction of FH was no significant difference (Fig. 2).

**Table 3:** Imaging outcomes of patients in the two groups.

Variable	F group (n = 44)	T group (n = 43)	*P* value
Pre. PS	2.98 ± 0.76	3.00 ± 0.72	.900
FU. PS	3.14 ± 0.59	3.49 ± 0.70*	.014^#^
Change. PS	-0.16 ± 0.96	0.49 ± 1.10	.004^#^
Pre. CA (°)	33.97 ± 6.72	34.18 ± 6.74	.885
FU. CA (°)	30.90 ± 6.40*	26.09 ± 5.52*	<.001^#^
Change. CA (°)	3.07 ± 1.49	8.09 ± 4.90	<.001^#^
Pre. FH (mm)	23.86 ± 1.23	23.36 ± 1.77	.129
FU. FH (mm)	23.76 ± 1.25	22.75 ± 1.73	.002^#^
Change. FH (mm)	0.10 ± 1.45	0.61 ± 2.07	.186
Pre. FA (mm^2^)	211.01 ± 12.47	210.09 ± 12.75	.735
FU. FA (mm^2^)	197.32 ± 13.92*	185.43 ± 20.24*	.002^#^
Change. FA (mm^2^)	13.69 ± 12.84	24.66 ± 18.50	.002^#^

PS = Pfirrmann score, CA = Cobb angle, FH = foraminal height, FA = foraminal area.

# Comparison of parameters between the two groups.

* *P* < .05, comparison of parameters within the same groups before surgery and final FU.

**Figure 3.**
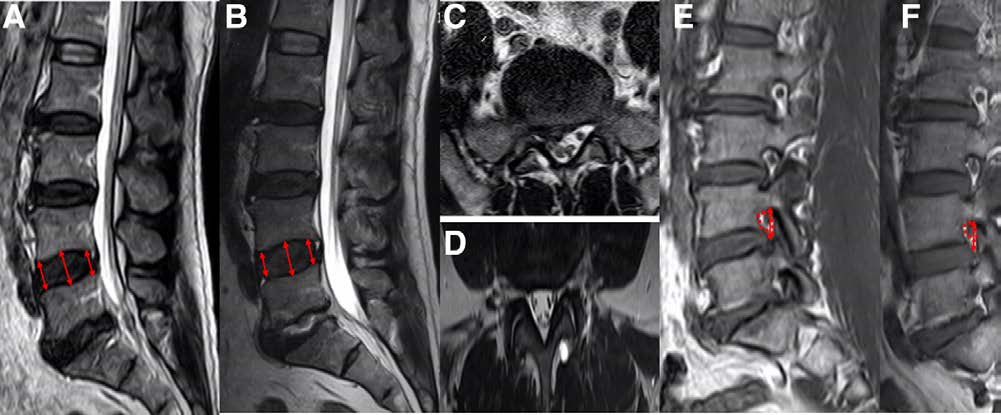
Typical case: A 50-year-old male patient presented with recurrent low back pain for 1 year, which was aggravated by numbness, pain and fatigue of the right lower limb for more than 1 month. Diagnosis: lumbar disc herniation (L5/S1). The pain symptoms after L5/S1 lumbar intervertebral discectomy under foraminal endoscopy were significantly relieved, and the improvement rate of JOA in the last FU was 79.23%. The changes of intervertebral disc height, intervertebral foramen height and area were not obvious. Preoperative magnetic resonance (ACE) final follow-up (BDF)).

The clinical results were summarized in Table 4. The JOA scores improved from 14.90 ± 2.20 to 24.12 ± 2.40 in F group, and 14.55 ± 1.98 to 25.71 ± 2.12 in T group. No significant difference was observed in JOA scores between the two groups at the final FU. There were 40 (90.90%) patients who acquired significant alleviation of original symptoms in group F, whereas in group T, 41(95.35%) patients had symptom alleviation. No difference was observed between the peri-operative complications, three patients in group T experienced surgery-related complications: one with lumbar hematoma, one with surgery site infection and one with cerebrospinal fluid leakage. In group F, the number of patients with complications was one: one with Cerebrospinal fluid leakage. all patients received timely symptomatic treatment and all were cured. The re-operation rates were 4.55% (2/44) in F group (two patients underwent open surgery because of the protruding of the operative segment.), and 2.33% (1/43) in T group (One patient underwent endoscopic revision because of ASD) at the final FU. Typical case: Figure 3.

**Table 4:** Clinical outcomes of patients in the two groups.

Variable	F group (n = 44)	T group (n = 43)	*P* value
Pre. JOA	14.90 ± 2.20	14.55 ± 1.98	.438
Post. JOA	24.12 ± 2.40	25.71 ± 2.12	.399
Final follow-up JOA	23.18 ± 2.35	24.01 ± 2.97	.078
Alleviation of original symptoms	40 (90.90%)	41 (95.35%)	.694
Re-operation rates	4.55% (2/44)	2.33% (1/43)	.000
Pulmonary embolism	0	0	
Root injury	0	0	
Cerebrospinal fluid leakage	1	1	
Lumbar hematoma	0	1	
Surgery site infection	0	1	
Total complications	1 (2.27%)	3(6.98%)	.592

## 4. Discussion

According to previous reports,^[7]^ the incidence of ASD after lumbar fusion surgery and non-fusion surgery was 5‐77% (mean 26.6%) and 10% respectively. However, the^[8,9]^ ASD after spinal fusion is considered to be multifactorial. Many literatures have reported that the degree of intervertebral disc degeneration was closely related to age (over 60 years old), genetic factors, high body mass index, preexisting stenosis or degeneration of adjacent segments, lumbar insufficiency, multi-segmental lumbar fixation and fusion.^[10–12]^ Therefore, this study aimed to eliminating interference of these factors, and there is no statistical difference of these factors between two groups before operation. In addition, the operation itself is also one of the important reasons resulting in ASD. Ekman et al found that lumbar fusion accelerated ASD. after long-term FU.^[13]^ Some scholars reported that the incidence of cephalic ASD examined by X-ray 2‐3 years after lumbar fixation and fusion was 38.5%.^[14]^ This study also focuses on the cephalic adjacent intervertebral disc. Radcliff et al pointed out that the rate of ASD after fusion was significantly higher than that in patients without decompression,^[15]^ and concluded that excessive distracting by the fusion cage to the intervertebral space was an important risk factor for ASD.^[16]^ In a retrospective study, Biden et al suggested that floating fusion, in which the lower end of the fusion vertebra located at L5, is a risk factor for ASD.^[9]^ In addition, floating fusion was more likely to develop ASD in patients with posterolateral lumbar fixation.^[17]^

Although various reasons were attributed to ASD from the different views of many studies, the author speculates that surgery-related biomechanical changes of the spine are one of the most reasonable mechanisms.

In 1983, Kirkaldy‐Willis put forward the theory of three-joint complex (composed of intervertebral disc and two posterior facet joints), and believed that this structure plays an important role in maintaining the stability of the spine.^[18]^ Liu et al, after six-year FU of patients accepting L4-5 fusion, found that the incidence of ASD was the highest in patients undergoing laminectomy.^[19]^ Imagama et al followed up 52 patients after L4-5 laminectomy or L4-5 fenestration fusion for five years, revealing that patients with fenestration were less likely to develop ASD.^[20]^ The results showed that the preservation of the structure of the posterior column of the lumbar spine is an important factor to avoid ASD. Lumbar fusion requires extensive peeling off of paraspinal muscles, removal of part of ligaments and bony structure, destruction of the stability of the three-joint complex, resulting in abnormal load distribution of the whole spine, prone to vertebral spondylolisthesis or fracture and other diseases.^[21]^ Therefore, it changes the original equilibrium relationship between the diseased vertebral body and the adjacent vertebral body, and aggravates the postoperative ASD.^[21–23]^ Ma et al found in the human cadaver model that the increase in stress on the facet joints after fusion may affect the degeneration of adjacent segments.^[24]^ Through the analysis of three-dimensional finite element model, the biomechanical load of the adjacent vertebral facet joint above the fusion segment is obviously abnormal.^[25,26]^

Makino at al reported that the incidence of ASD in 41 L4-5 PLIF patients with minimum intervertebral space distraction (12.2%) was significantly lower than that of previous ASD with PLIF distraction (31.8%).^[27]^ It is considered that the use of a smaller fusion cage to minimize the opening of the intervertebral space may prevent ASD. In a biomechanical study of a finite element model fused at the L4/5 level, stress on the L3/4 endplate and intervertebral disc increased during flexion/extension movement.^[28]^ In addition, in the cadaveric L3/4 fixation model, Cunningham et al observed an increase of pressure in the L2/3 intervertebral disc by 45% during flexion/straightening.^[29]^ It can be seen that the cadaveric experiment showed that the pressure in the proximal intervertebral disc of the adjacent intervertebral disc increased to a fixed level.^[29,30]^

Therefore, we think that the occurrence of ASD after fusion may be related to mechanical factors, the destruction and disorder of local structure, the range of motion of its upper adjacent segments and the compensatory load of facet joints.

Because the nucleus pulposus tissue is a colloidal semi-liquid substance with flow characteristics, the volume of the intervertebral disc will be further degraded and absorbed over time after nucleus pulposus resection.^[31]^ Therefore, the removal of the nucleus pulposus of PTED (percutaneous transforaminal endoscopic discectomy) leads to the decrease of the bearing capacity of intervertebral disc, which in turn leads to the decrease of the upper vertebral body. At the last FU, the height of the intervertebral space in the upper adjacent segment was lower than that before operation, and there was statistical significance (*P* < .05). It may be related to the natural process of aging. However, compared with TLIF, PTED can not only retain more spinal range of motion, but also retain as much intervertebral disc tissue as possible on the basis of ensuring the curative effect, which provides a pathological basis for self-repair and secondary stability in the later stage, and may reduce the incidence of ASD or delay the occurrence of ASD.

Many studies have shown that the decrease, disappearance or kyphosis of lumbar physiological curvature is closely related to the degeneration of intervertebral disc. Studies have shown that lumbar physiological curvature changes in patients with LDH may be the result of lumbar mechanical structural imbalance caused by lumbar degeneration.^[32]^ Hypolordosis in the instrumented segment increased the load on the posterior pedicle device, posterior shear, and strain on the vertebral plate at the adjacent level. Biomechanical effects may explain the long-term consequences of degenerative changes after lumbar fusion.^[33]^ The fusion of the lumbar spine in abnormal sagittal alignment and the loss of lumbar anterior convexity pre dispose the patient to degeneration of adjacent segments.^[34]^ In this study, the changes of adjacent segments and CA in group F were significantly lower than those in group T, indicating that PTED can maintain physiological curvature and mechanical balance of spinal structure to some extent, and reduce the incidence of lumbar disc height loss.

Intervertebral disc degeneration can directly and indirectly affect the area of intervertebral foramen. Cinotti et al found that intervertebral disc height loss can lead to intervertebral foramen stenosis by measuring 160 intervertebral foramen in dry cadaver specimens and 50 intervertebral foramina in fresh cadaveric spine.^[35]^ In this study, the cross-sectional area of intervertebral foramen decreased before operation and at the last FU, and the change in group F was lower than that in group T. The stenosis of intervertebral space caused by intervertebral disc degeneration can significantly reduce the height of intervertebral foramen, especially the minimum sagittal diameter of intervertebral foramen. It may be due to the natural degeneration of the intervertebral disc or the change of posture during the examination of the patient.

In the past, many scholars have shown that the foraminal endoscope had a definite effect for LDH in early stage, and could significantly improve the pain symptoms.^[36]^ In 588 patients with LDH treated by intervertebral foramen endoscopy and followed up for more than 2 years, the excellent and good recovery rate was 95.3%, and the recurrence rate was 3.6%.^[37]^ Studies have reported that a small number of ASD patients can progress to symptomatic ASD, and their imaging findings are not necessarily correlated with symptoms after spinal fusion. Therefore, there was no significant difference in the JOA scores between the two groups at the last FU in this study.^[38–40]^ At the same time, compared with open surgery, foraminal endoscopic surgery was performed under local anesthesia, the operator can observe patient’s feedback well, and there is no need to expose the herniated intervertebral disc by pulling the nerve root and dural sac during the operation, which reduces the risk of nerve injury.^[41]^ Therefore, this study also found that minimally invasive surgery has a low complication rate and a low recurrence rate.

### 4.1. Limitations

There may be differences in baseline characteristics of the preoperative population that affect the accuracy of this study. In addition, this is a small sample and short-term FU imaging measurement study that requires a large population, prospective study to analyze changes in adjacent segments with conservatively treated controls. Finally, the same team of physicians responsible for standardized procedures to assess FU symptoms and independent measurements of imaging parameters was blinded to this study.

## 5. Conclusion

Both TLIF and endoscopic surgery can achieve good results in the treatment of LDH, but the risk of lumbar disc height loss and intervertebral foramina reduction in the adjacent segment after intervertebral foraminal surgery is lower.

## Author contributions

SW analyzed and interpreted the patient data, and was a major contributor in writing the manuscript. DY and GZ performed the examination of the data, and substantively revised the manuscript, conducted the acquisition of data. JC and FZ proposed the idea of study design. JS and RY finished the final assessment of the manuscript. All authors read and approved the final manuscript.

**Data curation:** Deyu Yang, Feng Zhao, Shunmin Wang.

**Formal analysis:** Shunmin Wang.

**Funding acquisition:** Gengyang Zheng, Jiangang Shi, Ruijin You.

**Investigation:** Gengyang Zheng, Shunmin Wang.

**Methodology:** Ruijin You, Shunmin Wang.

**Software:** Ruijin You.

**Supervision:** Deyu Yang, Jiangang Shi, Jie Cao.

**Project administration:** Shunmin Wang.

**Validation:** Gengyang Zheng, Jie Cao, Ruijin You.

**Writing – original draft:** Shunmin Wang.

**Writing – review &amp; editing:** Jiangang Shi, Jie Cao, Shunmin Wang.
